# Beyond Smoking Prevalence: Exploring the Variability of Associations between Neighborhood Exposures across Two Nested Spatial Units and Two-Year Smoking Trajectory among Young Adults

**DOI:** 10.3390/ijerph13010106

**Published:** 2016-01-06

**Authors:** Adrian E. Ghenadenik, Katherine L. Frohlich, Lise Gauvin

**Affiliations:** 1 Département de Médecine Sociale et Préventive, École de Santé Publique (ESPUM), Université de Montréal, 7101, Av. du Parc, Montreal, QC H3N 1X9, Canada; katherine.frohlich@umontreal.ca (K.L.F.); lise.gauvin.2@umontreal.ca (L.G.); 2 Institut de Recherche en Santé Publique de l’Université de Montréal (IRSPUM), 7101, Av. du Parc, Montreal, QC H3N 1X9, Canada; 3 Centre de Recherche du Centre Hospitalier de l’Université de Montréal (CRCHUM), 850 Saint Denis Street, Montreal, QC H2X 0A9, Canada

**Keywords:** neighborhood exposures, smoking trajectory, young adults, spatial scale, multilevel

## Abstract

Young adults have the highest prevalence of smoking amongst all age groups. Significant uptake occurs after high school age. Although neighborhood exposures have been found to be associated with smoking behavior, research on neighborhood exposures and the smoking trajectories among young adults, and on the role of geographic scale in shaping findings, is scarce. We examined associations between neighborhood exposures across two nested, increasingly large spatial units and smoking trajectory over two years among young adults living in Montreal, Canada. A sample of 2093 participants aged 18–25 years from the Interdisciplinary Study of Inequalities in Smoking (ISIS) was surveyed. The dependent variable was self-reported smoking trajectory over the course of two years. Residential addresses, data on presence of tobacco retail outlets, and the presence of smoking accommodation facilities were coded and linked to spatial units. Three-level multinomial models were used to examine associations. The likelihood of being a smoker for 2+ years was significantly greater among those living in larger spatial unit neighborhoods that had a greater presence of smoking accommodation. This association was not statistically significant at the smaller spatial units. Our findings highlight the importance of studying young adults’ smoking trajectories in addition to static smoking outcomes, and point to the relevance of considering spatial scale in studies of neighborhoods and smoking.

## 1. Introduction

Smoking continues to be an unacceptable burden on the health of Canadians. Mortality caused by tobacco-related disease accounts for approximately 37,000 annual deaths in this country [1]. Tobacco use is thus the leading cause of preventable premature mortality [2]. This is of particular concern among young adults, who have the highest prevalence of smoking of all age groups: In 2012, prevalence in the 20–24 year and 25–34 year age groups was 17.9% and 18.5% respectively, compared to a prevalence of 14.6% in the general population [2].

Young adults not only have the highest prevalence, but they also experience significant changes in smoking behavior, both in terms of initiation and quit attempts [3,4,5]. Empirical studies provide evidence of significant smoking uptake in this age group, finding that as many as 25% of youth who took up smoking did so before age 24, but after high school age [3,4,6,7]. Moreover, the number of young adult smoking initiates appears to be surpassing that of adolescents, as suggested in a systematic review of smoking initiation in the United States and Canada [8]. These trends call for attention because young adulthood is recognized as a key transitional period in terms of health behaviors and risk factors, not only by public health organizations [9] but also by the tobacco industry, who identifies it as an important window to market tobacco products [10]. Nonetheless, this age group is frequently overlooked in tobacco control efforts [4] and underutilize evidence-based cessation treatments [11]. This, in spite of studies suggesting that successful prevention of smoking initiation during young adulthood increases the likelihood of never becoming a regular smoker [5].

Similar to other health outcomes, there are significant place-based inequalities in smoking [12,13,14,15,16,17,18,19,20,21,22,23,24,25,26,27,28]. For example, on the Island of Montreal, smoking rates differ significantly across place of residence; in 2007–2010, they ranged from 15.6% to 36.0% across the region’s Health and Social Services administrative units [29]. These studies highlight the potential role of neighborhood-level factors as enablers and barriers to health, above and beyond individual-level socio-demographic characteristics such as age, sex and education, well known correlates of smoking [30]. Differences in health behaviors and outcomes at the neighborhood level are thought to arise in part due to differential distributions of resources available to their residents [31], as well as their differential capability to access them [32]. These resources may be health-promoting (e.g., greater levels of neighborhood trust can be a protective factor for smoking behavior) [33] or health-deterring (e.g., a greater presence of tobacco retail stores may be associated with higher smoking rates) [34].

Acknowledging the importance of understanding the potential role of neighborhood exposures in shaping smoking behavior, there is a significant body of research devoted to the examination of associations between neighborhood-level exposures and smoking. Among an array of environmental exposures potentially associated with smoking outcomes, proximity and density of tobacco retail stores and presence of smoking accommodation facilities are thought to be relevant. A considerable amount of research has explored associations between smoking outcomes and proximity and density of tobacco retail stores. In adult populations, a greater density of tobacco outlets around address of residence has been found to be associated with a higher likelihood of smoking [14,35] and a lower likelihood of smoking cessation [36,37]. Among adolescents, proximity and a greater density of tobacco outlets around schools and homes have been found to be associated with higher experimental smoking rates [38], the number of cigarettes smoked daily [14,39], and likelihood of smoking [40]. However, while associations between exposure to tobacco retail and smoking outcomes have been explored in adolescents and adult populations, very few studies have examined the potential impact of proximity and density of tobacco retail stores on smoking outcomes in young adults. A recent study found a positive association between density of tobacco outlets and smoking initiation in individuals aged 18–34 years [41]. Another recent study included both residential neighborhoods and activity spaces as areas of exposure to density of tobacco outlets. Results showed a higher likelihood of smoking in young adults exposed to greater numbers of tobacco retail stores both around residential neighborhoods and activity spaces [42].

Moreover, limited research has been conducted to examine associations between the presence of smoking accommodation facilities and smoking outcomes, either in relationship to the implementation of smoking bans in public spaces or without such relationships. Following a national ban on indoor smoking in hospitality venues, a French study found that, while indoor smoking decreased significantly, the offer of outdoor smoking spaces increased. This resulted in reported rates of outdoor smoking increasing from 33.6% at baseline to 75.9% at follow-up, suggesting that, although overall individual prevalence declined, in many cases smoking simply moved outdoors [43]. Interestingly, a recent study examining associations between exposure to patio smoking and smoking cessation found that smokers who were exposed to smoking in patios were less likely to have attempted to cease smoking and more likely to relapse after having made a quit attempt [44]. These studies highlight the relevance of outdoor smoking accommodation facilities as a potential hurdle to smoking cessation. Although it is also possible that these spaces play a role in facilitating smoking initiation (e.g., through exposure to social cues), to date, no empirical studies have examined associations between exposure to smoking accommodation facilities and smoking initiation. Moreover, as with proximity and density of tobacco retail stores, very few studies (if any) have examined associations between the presence of smoking accommodation facilities and smoking outcomes in young adults.

Among a number of conceptual and methodological challenges that must be addressed in order to better understand associations between neighborhood exposures and health outcomes, one important consideration is the definition of appropriate spatial scales. Among the set of challenges related to the analysis of spatial data [45], this issue is central to studies of neighborhoods and health, given that processes underlying associations between neighborhood-level exposures and health outcomes are likely to operate at different scales depending on specific exposure-outcome pairs. For example, the geographic scale at which social networks and distance to retail outlets respectively impact smoking behavior may not be the same. Moreover, the potential impact of these two exposures may operate at different scales in the context of other health outcomes such as depression or cardiovascular disease. Therefore, the operationalization of spatial units must be specific to the associations under study [46,47,48]. Nonetheless, although some scholars have conducted studies to better understand the impact of using different spatial scale definitions in studies of neighborhoods and health [49,50,51,52], to our knowledge few studies of neighborhoods and smoking have examined the potential role of spatial scales in shaping research findings. Importantly, the choice of a spatial scale that does not correspond to the geographical level at which a specific exposure is hypothesized to influence smoking outcomes may translate into an inappropriate operationalization of geographical boundaries, which in turn may result in significant measurement error [53]. This issue is widely recognized in the literature as the “modifiable area unit problem” (MAUP) [54]. In the presence of MAUP, estimates of between-area variation may depend on how boundaries are defined [52,55] and may potentially result in two types of error. The first, usually called the “scale effect”, may arise when different statistical results are obtained depending on the scale at which data are aggregated. The second, usually called the “zoning effect”, may occur when different statistical results are obtained depending on how boundaries of a territory are defined [52,53]. Some scholars have called for the use of theories linking spatial scales, mediating processes, and health outcomes prior to the analysis of neighborhood effects [48,56]. Although it would be ideal to define spatial units based on theory, exploratory empirical analyses can allow for initial exploration of spatial issues in the absence of strong theory [57]. In keeping with this notion, the majority of studies of neighborhoods and smoking have used empirical approaches in order to examine associations between exposures and smoking outcomes rather than identifying theory-based spatial unit definitions. In general terms, two broad types of spatial units have been used in the literature. The first is a “proximal” unit type that seeks to capture the more immediate neighborhood environment, either by the use of administrative areas such as census tracts and municipal subdivisions or by the creation of buffer zones with different radii (see for example [22,28]). The second is a “community” unit type that captures environments relevant to health processes that likely operate at a larger scale, such as social norms and community practices (see for example [19,25]).

Another important issue for research on neighborhoods and smoking is the study of associations between exposures and changes in smoking behavior, or lack thereof. Research in this area has typically focused on static smoking variables as their outcome of interest. For example, all of the studies on smoking included in a recent systematic review of health-risk behaviors and neighborhood deprivation examined current smoking status as their outcome [58]. This was also the case for a vast array of studies of different neighborhood exposures and smoking behaviors including current smoking status, smoking initiation and smoking cessation (see for example [35,36,41,59]). Although it is certainly important to examine associations between neighborhood exposures and smoking outcomes using cross-sectional designs, this approach cannot provide information regarding the potential role of neighborhood exposures in influencing changes in smoking status, such as becoming a new smoker, becoming a new non-smoker, or lack of change in any direction. This is particularly important among young adults because this age group experiences significant changes in smoking status. Hammond (2005) proposes that, contrary to hypotheses postulating that smoking behavior becomes largely fixed after high school age, young adults display variable smoking patterns, with significant smoking uptake taking place after high school age, in many cases shifting repeatedly between daily and occasional smoking [60]. Therefore, regular smoking habits may only develop later in life [61]. Moreover, in comparison to older adults, young adults are more likely to attempt to quit smoking [11].

This variability in smoking behavior in young adults was portrayed in the results of a 2004 study of changes in smoking behavior over a four-year period among a cohort of college students. Within this timeframe, 13% of daily smokers at baseline ceased to smoke and 28% changed their status from daily to occasional smokers, 14% of occasional smokers at baseline became daily smokers, while 51% ceased to smoke, and 11% of non-smokers at baseline initiated occasional smoking. Additionally, 87% of smokers at baseline and 50% of occasional smokers at baseline continued to smoke after four years [62]. Given the significant differences in smoking behavior between college-educated and non-college-educated young adults in the United States [63], these results may not generalize beyond college-educated young adults. Nonetheless, they illustrate the instability of smoking status in this age group.

To date, the bulk of research on neighborhoods and smoking has mainly focused on how neighborhood exposures are associated with static smoking outcomes, such as smoking rates or cessation rates at a specific point in time. Moreover, the vast majority of these studies has devoted their efforts to adolescent and adult populations, while little attention has been paid to young adults. Therefore, it is crucial to understand the role of neighborhood-level exposures in shaping the smoking trajectories in this population.

Additionally, in spite of calls for explicit consideration of spatial scales more specific to the exposures and outcomes under examination, there is a paucity of studies exploring the variability of associations between neighborhood exposures and smoking across spatial scales. Hence, empirical research in this area can contribute to improving knowledge of associations between neighborhood-level exposures and the smoking trajectories in young adults.

The objective of this study was to examine associations between the presence of tobacco retail stores, the presence of smoking accommodation facilities, and self-reported smoking trajectory among young adults across two nested spatial units in Montreal, Canada. One specific question was asked: For what categories of smoking trajectory and at what spatial scales are these associations statistically significant? We hypothesized that individuals living in neighborhoods with a greater presence of tobacco retail outlets and/or a greater presence of smoking accommodation facilities would be: (1) more likely to change their status from non-smoker to smoker over two years, or to maintain their smoker status during this two-year period; and (2) less likely to change their smoking status from smoker to non-smoker over two years, or to maintain their non-smoker status during this two-year period.

## 2. Methods

### 2.1. Study Sample

This study was conducted in the context of the Interdisciplinary Study of Inequalities in Smoking (ISIS), a cohort study with the objective of better understanding the joint role of individual and neighborhood factors in producing social inequalities in smoking in Montreal among young adults [64]. Analyses were based on the ISIS baseline sample from 2011 to 2012. This sample was composed of non-institutionalized individuals aged 18–25 years, proficient in either English or French, who had been living at their current address for at least one year at time of first contact. An initial random sample of 6020 individuals obtained from Quebec’s provincial health insurance program was contacted through a nominalized letter between November 2011 and August 2012. At the end of the recruitment period, 349 individuals refused to participate, 458 were declared ineligible and 3111 could not be reached, resulting in a final sample of 2093 participants. The response rate was 37.6%. While relatively low, these rates are not uncommon in observational studies and could be attributed to unreported moves, inaccurate mailing addresses or to a lack of interest in participating in the study. Participants had similar characteristics to those of participants in the Canadian Community Health Survey (CCHS), a Statistics Canada survey designed to gather health-related data at the health region level [65]. They were, however, in slightly less good physical and mental health and included a lower proportion of daily smokers and a higher proportion of non-smokers. Full details on sampling and survey procedures are available elsewhere [64].

### 2.2. Description of Neighborhood Spatial Scales

To examine the potential role of different spatial scales in shaping findings regarding associations between neighborhood exposures and smoking trajectory over 2 years among young adults, we used two empirical spatial unit definitions. The first, Health and Social Services catchment areas, called Centres de santé et de services sociaux (CSSSs; *n* = 12, mean area = 41.7 km^2^, mean 2011 population = 157,210), may capture processes operating at a larger scale. CSSSs, created in 2004, are administratively-defined geographic service units comprising a local network of health and social services. Territories typically include several partners, notably general practitioners, pharmacies, community organizations, private organizations, specialized health and social services organizations (e.g., hospitals, youth services centres and rehabilitation centres), and multi-sectoral partners [66].

The second definition, sociological neighborhoods (SNs; *n* = 111, mean area = 4.5 km^2^, mean 2011 population = 17,000), may capture processes operating within the more immediate neighborhood environment. In contrast to CSSS territories, SNs are community-defined spatial units based both on administrative boundaries and shared perceptions of their residents regarding their history, sense of belonging, infrastructure and services, and population characteristics. These territories were created in 2008 by the Direction de santé publique de Montréal (Montreal’s Public Health Department) in partnership with local organizations to better capture the subjective sense of neighborhood experienced by residents. The 111 SNs on the Island of Montreal are nested within the 12 CSSSs and respect Statistics Canada’s census tract and dissemination area boundaries. These spatial units are fairly homogeneous in terms of socioeconomic characteristics and are recognized as relevant spatial divisions for local development initiatives [67]. Maps of the territories used for planning and delivery of health and social services on the Island of Montreal can be found elsewhere [68,69].

### 2.3. Measures of Neighborhood Exposure

Two exposure measures were used in this study. The first was presence of tobacco retail stores at each spatial unit. To construct this measure, data regarding retail outlets legally selling tobacco products were extracted from the 2011 DMTI Enhanced Points of Interest Database [70]. This database is a collection of geocoded address points covering a comprehensive range of land uses, including tobacco retail stores. This data source was validated using the 2010 database version, for convenience stores and supermarkets. Data were found to be adequately representative, with sensitivity values (the capacity to detect stores present in the field) of 0.59 and 0.75, and positive predictive values (the ability to list only stores that actually existed in the field) of 0.73 and 1.00 for convenience stores and supermarkets respectively [71].

To operationalize the presence of tobacco retail stores, the first step was to compute the number of retail outlets selling tobacco products for each dissemination area (DA) where at least one cohort participant resided. DAs are the smallest geographic census areas in Canada, composed of one or more adjacent dissemination blocks with a population of 400 to 700 persons. There are 3175 DAs on the Island of Montreal, with a mean area of 0.16 km^2^. A total of 1399 of the 3175 DAs were aggregated to create this exposure. Given that more than half of the DAs had no presence of tobacco retail stores (806 DAs, 57.6% of total), and most of those having presence of tobacco retail stores had only one outlet (369 DAs, 26.4% of total), the values obtained at the DA level were categorized as a dichotomous indicator of presence of at least one retail outlet selling tobacco products in the DA (0 = no presence). A similar approach has been used in previous studies (see, for example, [35]). Alternative approaches to the operationalization of this exposure were explored including density of tobacco retail outlets per 10,000 inhabitants and density of tobacco retail outlets per km^2^. Analyses revealed relatively low densities per 10,000 population compared to those reported in a recent British study [72]: 96% of the measured DAs had a density of 49.6 per 10,000 inhabitants or lower, the lowest reported value in this study. Since no locally validated standard measures of density of tobacco retail stores that account for population density were available, we chose to use a dichotomous indicator of presence of at least one retail outlet selling tobacco products in the DA.

Second, DA-level values were aggregated at each of the two spatial scales, CSSSs and SNs, and mean values of presence of tobacco retail stores were computed. Finally, in order to contrast neighborhoods with greater presence of this exposure against all other neighborhoods, these values were recoded into a dichotomous indicator. Spatial units at which the highest means were observed (the top quartile) were coded “1” to reflect greater presence, whereas all other areas were coded “0”, reflecting lesser presence of tobacco retail.

The second exposure measure was presence of smoking accommodation facilities. Data used to construct this indicator were extracted from the ISIS observational database. Observational data were collected between June and September 2012 using a validated observation grid [73]. To develop this grid, a theoretical framework [32] conceptualizing the nature of neighborhoods and their potential role in the generation of health inequalities was used. Based on this framework, 86 indicators were constructed, operationalizing a range of neighborhood environmental exposures (e.g., quality of the built environment, neighborhood disorder, presence of facilities accommodating smokers). These indicators were evaluated by trained raters who filled out a paper form. The instrument’s inter-rater reliability and temporal stability were assessed through a generalizability study showing that 75% of the indicators in the observation grid were measured with acceptable to excellent reliability [73]. A random sample of street segments (a portion of a street between two intersections, measuring more than 60 meters in length) within Montreal’s 35 local community service territories (CLSC) was selected (*n* = 1399). CLSC territories are administrative units based on the provision of health and social services created by the Ministry of Health and Social Services.

To operationalize the presence of smoking accommodation facilities, street sections having at least one smoking-accommodating feature (ashtrays in commercial outlets, institutions and community organizations, and terraces/patios in bars and restaurants) were coded “1” to indicate presence of such facilities, whereas street sections with no smoking accommodation facilities were coded “0”, indicating no presence. The majority of the DAs had no smoking accommodation facilities (1033 DAs, 73.8% of total DAs). DA-level values were aggregated at each of the two spatial scales, CSSSs and SNs, and mean values of presence of tobacco retail stores were computed. Lastly, in order to contrast neighborhoods with greater presence of this exposure against all other neighborhoods, these values were recoded into a dichotomous indicator of smoking accommodation facilities. Spatial units at which the highest means were observed (the top quartile) were coded “1” to reflect greater presence, whereas all other areas were coded “0”, reflecting lesser presence.

### 2.4. Outcome Measures

The dependent variable for this study was self-reported smoking trajectory. To measure this variable, the study population was classified into four categories. These categories were constructed based on self-reported smoking behavior at baseline, measured in 2011, and self-reported previous smoking behavior over a 2-year period. The cut-off point used to construct the categories of smoking trajectory was based on empirical studies of milestones of nicotine dependence and smoking cessation [74,75,76,77]. The following baseline ISIS individual survey questions were used in this study:

(1) Currently, do you smoke cigarettes every day, occasionally, or never?
(2) Have you ever smoked an entire cigarette?
(3) *How old were you when you smoked an entire cigarette for the first time?*
(4) When was the last time you smoked a cigarette?

Based on responses to these questions, each participant was allocated to one of four nominal categories. Participants who either never smoked an entire cigarette or reported a non-smoker status at baseline and smoking a cigarette for the last time 2 years ago or longer were categorized as non-smokers for 2 years or longer. Participants who reported an occasional or regular smoker status at baseline and smoking an entire cigarette for the first time 2 years ago or longer were categorized as smokers for 2 years or longer. Participants who reported an occasional or regular smoker status at baseline and smoking an entire cigarette for the first time less than 2 years ago were categorized as smokers for fewer than 2 years. Finally, participants who reported a non-smoker status at baseline and smoking a cigarette for the last time less than 2 years ago were categorized as non-smokers for fewer than 2 years.

### 2.5. Covariates

Three individual-level covariates were used for this study: age, sex, and educational attainment. All variables were dichotomized. Participants aged 18–21 years were coded “0”, while those aged 22–25 years were coded “1”. Male participants were coded “0”, whereas female participants were coded “1”. Finally, participants who completed high school or lower and were not enrolled in post-secondary studies at the time of survey were coded “0”, and those who attained at least a post-secondary degree or were enrolled in post-secondary studies at the time of survey were coded “1”.

Fully-adjusted models included a neighborhood socioeconomic position (SEP) variable to examine potential confounding of associations. Neighborhood SEP was operationalized using Pampalon’s material deprivation index quartiles (1 = least deprived, 4 = most deprived) [78]. This index is composed of three indicators: education, work status and income (proportion of persons who have no high-school diploma, the ratio of employment to population and average income), which are widely used as measures of SEP.

### 2.6. Statistical Analyses

Associations between neighborhood exposures and smoking trajectory were examined using three-level multinomial models: Level-1 = individuals, Level-2 = SNs, Level-3 = CSSSs. Multilevel models are widely used in studies of neighborhood effects, among other reasons, due to their ability to account for correlated or clustered observations and to describe the variability and heterogeneity in the population above and beyond average relationships [79]. Models were built using HLM V.7 software (Scientific Software International Inc.: Skokie, IL, USA), following a “step-up” approach, in which multilevel models are progressively specified. HLM is a statistical software designed to fit a variety of linear and non-linear models using hierarchically-structured data allowing for continuous, count, ordinal, and nominal outcome variables [80].

First, to explore the variance in smoking trajectory at each spatial unit, three-level intercepts-only models were built. Second, level-1 models were built to explain within-neighborhood variability, adding three socio-demographic covariates: age, sex, and educational attainment. Third, level-2 and level-3 models with no level-1 variables were constructed for each measure of exposure at both spatial levels. Finally, random-intercept level-2 and level-3 models including predictors at all levels were built.

## 3. Results

A total of 1183 female (56.5%) and 910 male (43.5%) young adults (YA) participated in the study. Approximately 51% of them were aged 18 to 21 years (49% were aged 22 to 25 years). The majority of participants (82.9%) completed or were pursuing post-secondary studies. Information regarding education was missing for 10 participants. The smoking status of the majority of young adults did not change over the two-year period. Most participants were non-smokers for two years or longer (1351 YA, 64.5% of the sample), while almost one-fifth were smokers for two years or longer (409 YA, 19.5% of the sample). 320 participants reported a change in their smoking status over a two-year period: 252 participants (12%) were non-smokers for fewer than two years (12% of the sample), while 68 participants (3.2%) were smokers for fewer than two years. Information regarding smoking trajectory was missing for 13 participants. Since missing data were not imputed, all analyses were conducted based on a subsample of 2070 participants, reflecting missing data for a total of 23 YA. At the SN level, 26 SNs (23.4%) had a greater presence of tobacco retail outlets, while 27 SNs (24.3%) had a greater presence of smoking accommodation facilities. At the CSSS level, three CSSSs (25%) had a greater presence of both exposures. Details appear in Table 1.

**Table 1:** Descriptive statistics of the analytical sample.

Variable	N (%)
**Age**	2093
18–21 years, (%)	1065 (50.9)
22–25 years, (%)	1028 (49.1)
**Sex**	2093
Male, (%)	910 (43.5)
Female, (%)	1183 (56.5)
**Education**	2093
High School or lower, (%)	347 (16.6)
CEGEP/Trade School or higher, (%)	1736 (82.9)
Missing data	10 (0.5)
**Smoking Trajectory**	2093
Non-Smoker ≥ 2 years, (%)	1351 (64.5)
Smoker ≥ 2 years, (%)	409 (19.5)
Non-Smoker < 2 years, (%)	252 (12.0)
Smoker < 2 years, (%)	68 (3.2)
Missing data	13 (0.6)
**Presence of Tobacco Retail Outlets—SN Level**	111
Lesser presence	85 (76.6)
Greater presence	26 (23.4)
**Presence of Smoking Accommodation Facilities—SN Level**	111
Lesser presence	84 (75.7)
Greater presence	27 (24.3)
**Presence of Tobacco Retail Outlets—CSSS Level**	12
Lesser presence	9 (75.0)
Greater presence	3 (25.0)
**Presence of Smoking Accommodation Facilities—CSSS Level**	12
Lesser presence	9 (75.0)
Greater presence	3 (25.0)

Intercepts-only models revealed significant between-area variance in smoking trajectory (Level-3 variance = 0.04027, *p*-value = 0.009) at the larger spatial unit definition (CSSSs). Conversely, no statistically significant between-area variance (Level-2 variance = 0.00019, *p*-value > 0.500) was observed at the smaller spatial units (SNs).

Three-level models with individual socio-demographic covariates as predictors showed a significant association between age and smoking trajectory. The likelihood of being a smoker for two years or longer was higher among participants aged 22–25 years *vs.* 18–21 years (OR = 1.48; 95% CI: 1.18, 1.86). The likelihood of being a smoker for fewer than two years was significantly lower among participants aged 22–25 years (OR = 0.24; 95% CI: 0.13, 0.45). Associations between sex and smoking trajectory showed that female young adults had a lower likelihood of being a smoker for two years or longer in comparison to male young adults (OR = 0.78; 95% CI: 0.63, 0.98). Associations between educational attainment and smoking trajectory were not statistically significant. Details appear in Table 2 below.

**Table 2:** Results of multinomial multilevel regression models predicting smoking trajectory over 2 years from individual-level exposures among 2070 young adults living in Montreal, Canada in 2011–2012.

Variable	Odds Ratio (95% CI)
**Age (reference 18–21 Years)**	
22–25 years—Smoker ≥ 2 years	1.48 (1.18–1.86) **
22–25 years—Non-Smoker < 2 years	1.05 (0.80–1.38)
22–25 years—Smoker < 2 years	0.24 (0.13–0.45) **
22–25 years—Non-Smoker ≥ 2 years	Reference
**Sex (reference male)**	
Female—Smoker ≥ 2 years	0.78 (0.63–0.98) *
Female—Non-Smoker < 2 years	0.95 (0.72–1.25)
Female—Smoker < 2 years.	0.65 (0.40–1.07)
Female—Non-Smoker ≥ 2 years	Reference
**Education (reference completed high school or lower)**	
Completed/currently CEGEP/Trade School or higher—Smoker ≥ 2 years	1.19 (0.87–1.62)
Completed/currently CEGEP/Trade School or higher—Non-Smoker < 2 years	0.86 (0.61–1.22)
Completed/currently CEGEP/Trade School or higher—Smoker < 2 years	0.69 (0.38–1.26)
Completed/currently CEGEP/Trade School or higher—Non-Smoker ≥ 2 years	Reference

** *p*-value < 0.01; * *p*-value < 0.05.

Multilevel models with SN-level exposure variables as predictors showed a statistically significant association between a greater presence of tobacco retail stores and the likelihood of being a smoker for two years or longer. Individuals residing in sociological neighborhoods with greater presence of tobacco retail stores had a greater likelihood of being a smoker for two years or longer (OR = 1.56; 95% CI: 1.20, 2.05). Associations between a greater presence of smoking accommodation facilities at the SN level and smoking trajectory were not statistically significant.

Different from models with SN-level exposures as predictors, models with CSSS-level exposures as predictors showed a significantly higher likelihood of being a smoker for two years or longer for individuals living in CSSSs with a greater presence of smoking accommodation facilities (OR = 1.77; 95% CI: 1.35, 2.33). In contrast, associations between a greater presence of tobacco retail stores at the CSSS level and smoking trajectory were not statistically significant. Results of models predicting smoking trajectory from SN-level exposures and CSSS-level exposures appear in Table 3 and Table 4 respectively.

**Table 3:** Results of multinomial multilevel regression models predicting smoking trajectory over 2 years from SN-level exposures among 2070 adults living in Montreal, Canada in 2011–2012.

Variable	Odds Ratio (95% CI)
**Presence of Tobacco Retail Stores (Reference lesser presence)**	
Greater presence—Smoker ≥ 2 years	1.56 (1.20–2.05) **
Greater presence—Non-Smoker < 2 years	0.84 (0.58–1.22)
Greater presence—Smoker < 2 years	1.46 (0.82–2.61)
Greater presence—Non-Smoker ≥ 2 years	Reference
**Presence of Smoking Accommodation Facilities (reference lesser presence)**	
Greater presence—Smoker ≥ 2 years	1.12 (0.86–1.46)
Greater presence—Non-Smoker < 2 years	1.07 (0.80–1.45)
Greater presence—Smoker < 2 years	0.67 (0.37–1.23)
Greater presence—Non-Smoker ≥ 2 years	Reference

** *p*-value < 0.01.

Given the statistically significant associations between SN-level greater presence of tobacco retail stores, CSSS-level greater presence of smoking accommodation facilities and the likelihood of being a smoker for two years or longer, a first fully-adjusted model, “Model 1”, using these two exposures, as well as age and sex as individual socio-demographic predictors was tested. In this model, associations between age, sex, and the likelihood of being a smoker for two years or longer remained statistically significant. Interestingly, while the association between CSSS-level greater presence of smoking accommodation facilities and smoking trajectory over two years also remained statistically significant, this was not the case for SN-level greater presence of tobacco retail stores.

To examine whether the association between the presence of smoking accommodation facilities and smoking trajectory was confounded by neighborhood socioeconomic position (SEP), a second fully-adjusted model, “Model 2”, using neighborhood-level material deprivation was tested. After adjusting for material deprivation, CSSS-level presence of smoking accommodation facilities remained statistically significant, therefore suggesting no confounding of this association by neighborhood SEP. Results for these two models appear in Table 5.

**Table 4:** Results of multinomial multilevel regression models predicting smoking trajectory over 2 years from CSSS-level exposures among 2070 adults living in Montreal, Canada in 2011–2012.

Variable	Odds Ratio (95% CI)
**Presence of Smoking Accommodation Facilities (reference lesser presence)**	
Greater presence—Smoker ≥ 2 years	1.77 (1.35–2.33) **
Greater presence—Non-Smoker < 2 years	1.04 (0.76–1.43)
Greater presence—Smoker < 2 years	1.46 (0.85–2.50)
Greater presence—Non-Smoker ≥ 2 years	Reference
**Presence of Tobacco Retail Stores (reference lesser presence)**	
Greater presence—Smoker ≥ 2 years	1.35 (0.93–1.97)
Greater presence—Non-Smoker < 2 years	1.23 (0.91–1.65)
Greater presence—Smoker < 2 years	1.46 (0.87–2.45)
Greater presence—Non-Smoker ≥ 2 years	Reference

** *p*-value < 0.01.

**Table 5:** Results of fully-adjusted multinomial multilevel regression models predicting smoking trajectory over 2 years among 2070 adults living in Montreal Canada in 2011–2012.

Variable	Model 1	Model 2
	Odds Ratio (95% CI)	
**Age (reference 18–21 years)**		
22–25 years —Smoker ≥ 2 years	1.44 (1.15–1.81) **	1.48 (1.18–1.86s) **
22–25 years—Non-Smoker < 2 years	1.06 (0.81–1.39)	1.05 (0.80–1.37)
22–25 years—Smoker < 2 years	0.23 (0.12–0.43) **	0.23 (0.12–0.44) **
22–25 years—Non-Smoker ≥ 2 years	Reference	Reference
**Sex (reference male)**		
Female—Smoker ≥ 2 years	0.78 (0.62–0.98) *	0.77 (0.61–0.96) *
Female—Non-Smoker < 2 years	0.95 (0.72–1.25)	0.94 (0.71–1.24)
Female—Smoker < 2 years	0.65 (0.40–1.07)	0.65 (0.40–1.08)
Female—Non-Smoker ≥ 2 years	Reference	Reference
**SN-Level Presence of Tobacco Retail Stores (reference lesser presence)**		
Greater presence—Smoker ≥ 2 years	1.24 (0.92–1.67)	-
Greater presence—Non-Smoker < 2 years	0.79 (0.53–1.19)	-
Greater presence—Smoker < 2 years	1.41 (0.74–2.68)	-
Greater presence—Non-Smoker ≥ 2 years	Reference	-
**CSSS-Level Presence of Smoking Accommodation Facilities (reference lesser presence)**		
Greater presence—Smoker ≥ 2 years	1.59 (1.18–2.15) **	1.51 (1.08–2.11) *
Greater presence—Non-Smoker < 2 years	1.12 (0.79–1.58)	0.83 (0.57–1.20)
Greater presence—Smoker < 2 years	1.44 (0.79–2.62)	1.46 (0.77–2.77)
Greater presence—Non-Smoker ≥ 2 years	Reference	Reference
**Material Deprivation Quartile**		
Material Deprivation Quartile—Smoker ≥ 2 years	-	1.20 (0.89–1.61)
Material Deprivation Quartile—Non-Smoker < 2 years	-	1.43 (1.04–1.95) *
Material Deprivation Quartile—Smoker < 2 years	-	1.21 (0.69–2.13)
Material Deprivation Quartile—Non-Smoker ≥ 2 years	-	Reference

** *p*-value < 0.01; * *p*-value < 0.05.

## 4. Discussion

This study examined associations between the presence of tobacco retail stores, the presence of smoking accommodation facilities, and self-reported smoking trajectory over two years among young adults across two nested spatial units in Montreal, Canada. Results showed that the likelihood of being a smoker for two years or longer was higher among adults living in CSSSs where there was a greater presence of smoking accommodation facilities. Conversely, this association was not statistically significant at the SN level. In contrast, the likelihood of being a smoker for two years or longer was significantly higher among residents of neighborhoods with a greater tobacco retail presence at the smaller spatial unit, but this association was not statistically significant in fully-adjusted models. These results indicate that greater CSSS-level presence of smoking accommodation facilities is associated with being a persistent smoker over a two-year period, above and beyond individual socio-demographic characteristics. Additional analyses revealed that this association was not confounded by neighborhood-level socioeconomic position.

One mechanism that could explain the association between CSSS-level presence of smoking accommodation facilities and the greater likelihood of being a smoker for two years or longer is an increase in access and opportunities to smoke in hospitality venues and public places [43,81]. Evidence regarding smoke-free policies suggests that removing these environmental features contributes to curbing smoking prevalence by reducing smoking opportunities and by de-normalizing smoking [82]. Another potential mechanism underlying associations between the presence of smoking accommodation facilities and persistent smoking in young adults is the exposure to social, visual, and olfactory cues. These cues have been found to be associated with a lower intent to quit and a higher risk of relapse [83,84,85,86].

To date, most tobacco control interventions targeting this exposure have resorted to indoor smoking bans in public places, while outdoor smoking restrictions are frequently less stringent, allowing smoking in places such as outdoor patios and terraces. Moreover, these bans for the most part appear to have protected non-smokers from being exposed to second-hand smoke. Studies have shown that smokers tend to more frequently visit outdoor smoking venues [44], and report smoking more cigarettes in these places, in particular among younger individuals [87]. Therefore, future interventions may want to consider an extension of such bans to include outdoor public places, such as hospitality venues. The importance of extending smoking bans to outdoor spaces has been recognized by the Government of Quebec, who recently passed a bill prohibiting smoking in a variety of outdoor spaces including patios and terraces [88].

No significant associations between the two neighborhood exposures and other categories of smoking trajectory over two years were found. In the case of changes leading to a non-smoker status at baseline (non-smokers for two years or longer and non-smokers for fewer than two years), a lack of significant associations with a greater presence of tobacco retail outlets and a greater presence of smoking accommodation facilities appears to be a reasonable expectation. This is notable because these exposures were operationalized to detect associations with the greatest levels of exposure (*i.e.*, highest quartile of exposure *vs.* all other quartiles). The reverse may have resulted in findings of significant associations between the lowest levels of exposure and the likelihood of being a non-smoker at baseline. A greater presence of tobacco retail outlets is likely to increase accessibility to tobacco products [89] and exposure to point-of-sale marketing [90,91,92], while, as discussed above, it could be hypothesized that a greater presence of smoking accommodation facilities increases not only opportunities to smoke in public venues, but also social acceptance and exposure to social, visual and olfactory stimuli, all of which can trigger smoking. Therefore, it is more likely that greater levels of these exposures are associated with changes leading to a smoking status at baseline rather than the reverse.

While it is possible for a greater presence of tobacco retail outlets and smoking accommodation facilities to be associated with the likelihood of being a smoker for less than two years, the lack of statistically significant associations with this category of smoking trajectory over two years may suggest that these exposures are not sufficient to contribute to smoking initiation. A similar absence of statistically significant associations was found in a recent study of incidence and determinants of smoking initiation among young adults that examined three neighborhood-level exposures (tolerance of smoking around corner stores, around schools and around restaurants) likely to operate through mechanisms similar to those thought to underlie associations between the presence of tobacco retail outlets, smoking accommodation facilities and smoking trajectory over two years among young adults [4].

Another potential explanation for the absence of statistically significant associations in three of the four categories examined in this study is the instability of smoking status in this age group. Young adulthood is increasingly recognized as a crucial developmental period during which a number of important changes take place, including those related to health behaviors [9]. Since young adults may experience frequent changes from smoking to non-smoking and vice versa, it is possible that measurement at baseline captured one of these changes, albeit not with enough time for them to become more solidly established behaviors. Repeated measures designs may be helpful in addressing this issue.

The results of this study echo theoretical propositions regarding the specificity of scales at which health-related processes take place. As Diez-Roux (2007) proposes, “it is very plausible that areas of different size could be relevant for different processes and different health outcomes” [57] (p. 18). Therefore, as suggested by Gauvin *et al.* (2007), the use of an exposure-specific and outcome-specific spatial scale approach is likely to be best suited to the study of neighborhood effects on health [93]. The significant association between a greater presence of tobacco retail at the SN level (rather than at the CSSS level) and the likelihood of being a smoker for two years or longer suggests that geographic proximity may play an important role in facilitating access to tobacco products. This is in line with findings in the literature [36,37,94,95]. However, evidence of mechanisms underlying the association of presence of smoking accommodation facilities at the CSSS level rather than at the SN level are currently lacking. Future studies are warranted to explore this issue further.

In sum, two important issues regarding neighborhood-level exposures and smoking in young adults are highlighted in this study. First, differences in associations between neighborhood-level exposures and the likelihood of being a smoker for two years or longer suggest that additional knowledge regarding smoking outcomes in young adults can be gained by examining not only smoking prevalence, but also how this population goes through different stages of smoking behavior. Specific to this study, results suggest that variability in smoking trajectory over two years among young adults is explained, at least in part, by a greater CSSS-level presence of smoking accommodation facilities. Second, as evidenced by the differences in magnitude of associations between the specific exposures examined in this study and smoking trajectory over two years depending on the geographic scale of analysis, these results suggest that there is a need to consider spatial unit definitions appropriate to the specific exposure-outcome associations under analysis. Doing so is likely to contribute to the reduction of measurement error due to an inadequate operationalization of spatial scales.

This study also has a number of limitations. First, given the cross-sectional nature of its design, it was not possible to establish causal links between a greater CSSS-level presence of smoking accommodation facilities and the likelihood of being a smoker for two years or longer. Second, there is potential for selection bias, given the relatively low response rate (37.6%) to the individual questionnaire. Of note, non-responders were more likely to be male and to reside in most-deprived areas than responders. Third, even though the data source used to measure presence of tobacco retail outlets was validated and found to be adequately representative, misclassification bias cannot be ruled out. Fourth, given that data regarding presence of smoking accommodation facilities were collected at the dissemination area level, there is potential for understatement of this exposure in neighborhoods for which a more limited number of observations were conducted (e.g., in larger-area neighborhoods where no commercial street sections were observed). A larger sample could help examine this issue more thoroughly. Finally, neighborhood exposures were measured only once, and in consequence any changes that may have occurred were not taken into account. Given that changes in neighborhood-level exposures may be associated with the smoking trajectory, future studies could focus on the examination of changes in exposures across time.

## 5. Conclusions

Our findings highlight the importance of studying not only static smoking outcome measures such as smoking prevalence at a specific time-point, but also the smoking trajectory over two years. This is particularly important in young adults who experience repeated changes in smoking behavior throughout this life stage. Additionally, in line with calls for greater specificity in neighborhood effects studies [46,47,48,96], our results point to the relevance of spatial scale considerations in the studies of neighborhoods and smoking. Scale-dependent differences in associations between the two exposures examined in this study, and smoking trajectory over two years above and beyond individual socio-demographic characteristics, suggest that processes related to smoking in young adults take place at different scales and differ as a function of the specific exposures and outcomes being examined. Further research on specific neighborhood exposures and smoking trajectories in young adults is warranted.

## References

[1] Baliunas D., Patra J., Rehm J., Popova S., Kaiserman M., Taylor B. (2007). Smoking-attributable mortality and expected years of life lost in Canada 2002: Conclusions for prevention and policy. Chronic Dis. Can..

[2] Reid J.L., Hammond D., Rynard V.L., Burkhalter R. (2015). Tobacco Use in Canada: Patterns and Trends, 2015 Edition.

[3] Bernat D.H., Klein E.G., Forster J.L. (2012). Smoking Initiation During Young Adulthood: A Longitudinal Study of a Population-Based Cohort. J. Adolesc. Health.

[4] O’Loughlin J.L., Dugas E.N., O’Loughlin E.K., Karp I., Sylvestre M.P. (2014). Incidence and Determinants of Cigarette Smoking Initiation in Young Adults. J. Adolesc. Health.

[5] Tjora T., Hetland J., Aaro L.E., Wold B., Overland S. (2012). Late-onset smokers: How many, and associations with health behaviours and socioeconomic status. Scand. J. Public Health.

[6] Myers M.G., Doran N.M., Trinidad D.R., Wall T.L., Klonoff E.A. (2009). A Prospective Study of Cigarette Smoking Initiation During College: Chinese and Korean American Students. Health Psychol..

[7] Tercyak K.P., Rodriguez D., Audrain-McGovern J. (2007). High School Seniors’ Smoking Initiation and Progression 1 Year After Graduation. Am. J. Public Health.

[8] Freedman K.S., Nelson N.M., Feldman L.L. (2011). Smoking Initiation among Young Adults in the United States and Canada, 1998–2010: A Systematic Review. Prev. Chronic Dis..

[9] Bonnie R.J., Stroud C., Breiner H. (2015). Investing in the Health and Well-Being of Young Adults.

[10] Ling P.M., Glantz S.A. (2002). Why and How the Tobacco Industry Sells Cigarettes to Young Adults: Evidence From Industry Documents. Am. J. Public Health.

[11] Suls J.M., Luger T.M., Curry S.J., Mermelstein R.J., Sporer A.K., An L.C. (2012). Efficacy of Smoking-Cessation Interventions for Young Adults. Am. J. Prev. Med..

[12] Barnett R., Pearce J., Moon G. (2009). Community inequality and smoking cessation in New Zealand, 1981–2006. Soc. Sci. Med..

[13] Chahine T., Subramanian S.V., Levy J.I. (2011). Sociodemographic and geographic variability in smoking in the U.S.: A multilevel analysis of the 2006–2007 Current Population Survey, Tobacco Use Supplement. Soc. Sci. Med..

[14] Chuang Y.C., Cubbin C., Ahn D., Winkleby M.A. (2005). Effects of neighborhood socioeconomic status and convenience store concentration on individual level smoking. J. Epidemiol. Community Health.

[15] Datta G.D., Subramanian S.V., Colditz G.A., Kawachi I., Palmer J.R., Rosenberg L. (2006). Individual, neighborhood, and state-level predictors of smoking among US Black women: A multilevel analysis. Soc. Sci. Med..

[16] Duncan C., Jones K., Moon G. (1999). Smoking and deprivation: Are there neighborhood effects?. Soc. Sci. Med..

[17] Ellaway A., Macintyre S. (2009). Are perceived neighborhood problems associated with the likelihood of smoking?. J. Epidemiol. Community Health.

[18] Frohlich K.L., Potvin L., Gauvin L., Chabot P. (2002). Youth smoking initiation: Disentangling context from composition. Health Place.

[19] Hatzenbuehler M.L., Wieringa N.F., Keyes K.M. (2011). Community-Level Determinants of Tobacco Use Disparities in Lesbian, Gay, and Bisexual Youth: Results From a Population-Based Study. Arch. Pediatr. Adolesc. Med..

[20] Leatherdale S.T., Strath J.M. (2007). Tobacco Retailer Density Surrounding Schools and Cigarette Access Behaviors among Underage Smoking Students. Ann. Behav. Med..

[21] Lin E.Y., Witten K., Casswell S., You R.Q. (2012). Neighborhood matters: Perceptions of neighborhood cohesiveness and associations with alcohol, cannabis and tobacco use. Drug Alcohol. Rev..

[22] Miles R. (2006). Neighborhood disorder and smoking: Findings of a European urban survey. Soc. Sci. Med..

[23] Reijneveld S.A. (1998). The impact of individual and area characteristics on urban socioeconomic differences in health and smoking. Int. J. Epidemiol..

[24] Ross C.E. (2000). Walking, exercising, and smoking: Does neighborhood matter?. Soc. Sci. Med..

[25] Siahpush M., Borland R., Taylor J., Singh G.K., Ansari Z., Serraglio A. (2006). The association of smoking with perception of income inequality, relative material well-being, and social capital. Soc. Sci. Med..

[26] Smith K.C., Stillman F., Bone L., Yancey N., Price E., Belin P., Kromm E.E. (2007). Buying and Selling Loosies in Baltimore: The Informal Exchange of Cigarettes in the Community Context. J. Urban Health.

[27] Turrell G., Hewitt B.A., Miller S.A. (2012). The influence of neighborhood disadvantage on smoking cessation and its contribution to inequalities in smoking status. Drug Alcohol. Rev..

[28] Van Lenthe F.J., Mackenbach J.P. (2006). Neighborhood and individual socioeconomic inequalities in smoking: The role of physical neighborhood stressors. J. Epidemiol. Community Health.

[29] Tessier S., Drouin M., Simoneau M.E. (2013). Montréal sans Tabac: Plan de Lutte Contre le Tabagisme 2012–2015.

[30] Ross N.A., Tremblay S.S., Graham K. (2004). Neighborhood influences on health in Montreal, Canada. Soc. Sci. Med..

[31] Macintyre S., Ellaway A., Berkman L.F., Kawachi I. (2000). Ecological Approaches: Rediscovering the Role of the Physical and Social Environment. Social Epidemiology.

[32] Bernard P., Charafeddine R., Frohlich K.L., Daniel M., Kestens Y., Potvin L. (2007). Health inequalities and place: A theoretical conception of neighborhood. Soc. Sci. Med..

[33] Kandula N.R., Wen M., Jacobs E.A., Lauderdale D.S. (2009). Association Between Neighborhood Context and Smoking Prevalence Among Asian Americans. Am. J. Public Health.

[34] Pearce J., Hiscock R., Moon G., Barnett R. (2009). The neighborhood effects of geographical access to tobacco retailers on individual smoking behaviour. J. Epidemiol. Community Health.

[35] Marashi-Pour S., Cretikos M., Lyons C., Rose N., Jalaludin B., Smith J. (2015). The association between the density of retail tobacco outlets, individual smoking status, neighborhood socioeconomic status and school locations in New South Wales, Australia. Spat. Spatiotemporal Epidemiol..

[36] Halonen J.I., Kivimäki M., Kouvonen A., Pentti J., Kawachi I., Subramanian S.V., Vahtera J. (2013). Proximity to a tobacco store and smoking cessation: A cohort study. Tob. Control.

[37] Reitzel L.R., Cromley E.K., Li Y., Cao Y., Dela Mater R., Mazas C.A., Cofta-Woerpel L., Cinciripini P.M., Wetter D.W. (2011). The Effect of Tobacco Outlet Density and Proximity on Smoking Cessation. Am. J. Public Health.

[38] McCarthy W.J., Mistry R., Lu Y., Patel M., Zheng H., Dietsch B. (2009). Density of Tobacco Retailers Near Schools: Effects on Tobacco Use among Students. Am. J. Public Health.

[39] Scully M., McCarthy M., Zacher M., Warne C., Wakefield M., White V. (2013). Density of tobacco retail outlets near schools and smoking behaviour among secondary school students. Aust. N. Z. J. Public Health.

[40] Shortt N.K., Tisch C., Pearce J., Richardson E.A., Mitchell R. (2014). The density of tobacco retailers in home and school environments and relationship with adolescent smoking behaviours in Scotland. Tob. Control.

[41] Cantrell J., Pearson J.L., Anesetti-Rothermel A., Xiao H., Kirchner T.R., Vallone D. (2015). Tobacco Retail Outlet Density and Young Adult Tobacco Initiation. Nicotine Tob. Res..

[42] Shareck M., Kestens Y., Vallée J., Datta G., Frohlich K.L. (2015). The added value of accounting for activity space when examining the association between tobacco retailer availability and smoking among young adults. Tob. Control.

[43] Kennedy R.D., Behm I., Craig L., Thompson M.E., Fong G.T., Guignard R., Beck F. (2012). Outdoor smoking behaviour and support for outdoor smoking restrictions before and after France’s national smoking ban. Eur. J. Public Health.

[44] Chaiton M., Diemert L., Zhang B., Kennedy R.D., Cohen J.E., Bondy S.J., Ferrence R. (2014). Exposure to smoking on patios and quitting: A population representative longitudinal cohort study. Tob. Control.

[45] Fotheringham S.A., Rogerson P.A. (1993). GIS and spatial analytical problems. Int. J. Geogr. Inf. Syst..

[46] Diez Roux A.V. (2001). Investigating Neighborhood and Area Effects on Health. Am. J. Public Health.

[47] Macintyre S., Ellaway A., Cummins S. (2002). Place effects on health: How can we conceptualise, operationalise and measure them?. Soc. Sci. Med..

[48] Messer L.C. (2007). Invited Commentary: Beyond the Metrics for Measuring Neighborhood Effects. Am. J. Epidemiol..

[49] Cockings S., Martin D. (2005). Zone design for environment and health studies using pre-aggregated data. Soc. Sci. Med..

[50] Haynes R., Daras K., Reading R., Jones A. (2007). Modifiable neighborhood units, zone design and residents’ perceptions. Health Place.

[51] Krieger N., Chen J.T., Waterman P.D., Soobader M.J., Subramanian S.V., Carson R. (2002). Geocoding and Monitoring of US Socioeconomic Inequalities in Mortality and Cancer Incidence: Does the Choice of Area-based Measure and Geographic Level Matter?: The Public Health Disparities Geocoding Project. Am. J. Epidemiol..

[52] Schuurman N., Bell N., Dunn J.R., Oliver L. (2007). Deprivation indices, population health and geography: An evaluation of the spatial effectiveness of indices at multiple scales. J. Urban Health.

[53] Osypuk T., Galea S., Galea S. (2007). What Level Macro? Choosing Appropriate Levels to Assess How Place Influences Population Health. Macrosocial Determinants of Population Health.

[54] Openshaw S. (1984). The Modifiable Areal Unit Problem. Concepts and Techniques in Modern Geography.

[55] Stafford M., Duke-Williams O., Shelton N. (2008). Small area inequalities in health: Are we underestimating them?. Soc. Sci. Med..

[56] O’Campo P. (2003). Invited Commentary: Advancing Theory and Methods for Multilevel Models of Residential Neighborhoods and Health. Am. J. Epidemiol..

[57] Diez Roux A.V. (2007). Neighborhoods and health: Where are we and were do we go from here?. Rev. Epidemiol. Sante Publique.

[58] Algren M.H., Bak C.K., Berg-Beckhoff G., Andersen P.T. (2015). Health-Risk Behaviour in Deprived Neighborhoods Compared with Non-Deprived Neighborhoods: A Systematic Literature Review of Quantitative Observational Studies. PLoS ONE.

[59] Brown Q.L., Milam A.J., Smart M.J., Johnson R.M., Linton S.L., Furr-Holden C.D., Ialongo N.S. (2014). Objective and perceived neighborhood characteristics and tobacco use among young adults. Drug Alcohol Depend..

[60] Hammond D. (2005). Smoking behaviour among young adults: Beyond youth prevention. Tob. Control.

[61] Lantz P.M. (2003). Smoking on the rise among young adults: Implications for research and policy. Tob. Control.

[62] Wetter D.W., Kenford S.L., Welsch S.K., Smith S.S., Fouladi R.T., Fiore M.C., Baker T.B. (2004). Prevalence and predictors of transitions in smoking behavior among college students. Health Psychol..

[63] Green M.P., McCausland K.L., Xiao H., Duke J.C., Vallone D.M., Healton C.G. (2007). A Closer Look at Smoking among Young Adults: Where Tobacco Control Should Focus Its Attention. Am. J. Public Health.

[64] Frohlich K.L., Shareck M., Vallee J., Abel T., Agouri R., Cantinotti M., Daniel M., Dassa C., Datta G., Gagne T. (2015). Cohort Profile: The Interdisciplinary Study of Inequalities in Smoking (ISIS). Int. J. Epidemiol..

[65] Statistics Canada Canadian Community Health Survey—Annual Component (CCHS). http://www23.statcan.gc.ca/imdb/p2SV.pl?Function=getSurvey&SDDS=3226.

[66] Santé et Services Sociaux Québec Health and Social Services System in Brief. http://www.msss.gouv.qc.ca/en/sujets/organisation/en-bref/gouvernance-et-organisation/reseaux-locaux-de-services.

[67] Collectif Quartier Atlas Santé Montréal. http://www.collectifquartier.org/carte/atlas-sante-montreal/.

[68] Gouvernement du Québec, Espace Montréalais d’Information sur la Santé (EMIS) Territoires sociosanitaires à Montreal. http://emis.santemontreal.qc.ca/fileadmin/emis/Outil/Atlas/carte_pdf/decoupages/CSSS_CLSC_Voisinages_Montreal_01.pdf.

[69] Gouvernement du Québec, Espace Montréalais d’Information sur la Santé (EMIS) Les Voisinages de Montréal. http://emis.santemontreal.qc.ca/fileadmin/emis/Outil/Atlas/carte_pdf/decoupages/voisinage.pdf.

[70] DMTI Spatial Inc. (2011). Enhanced Points of Interest v.3 [Computer File].

[71] Clary C., Kestens Y. (2013). Field validation of secondary data sources: A novel measure of representativity applied to a Canadian food outlet database. Int. J. Behav. Nutr. Phys. Act..

[72] Shortt N.K., Tisch C., Pearce J., Mitchell R., Richardson E.A., Hill S., Collin J. (2015). A cross-sectional analysis of the relationship between tobacco and alcohol outlet density and neighborhood deprivation. BMC Public Health.

[73] Shareck M., Dassa C., Frohlich K.L. (2012). Improving the measurement of neighborhood characteristics through systematic observation: Inequalities in smoking as a case study. Health Place.

[74] Dierker L., He J., Kalaydjian A., Swendsen J., Degenhardt L., Glantz M., Conway K., Anthony J., Chiu W.T., Sampson N.A. (2008). The Importance of Timing of Transitions for Risk of Regular Smoking and Nicotine Dependence. Ann. Behav. Med..

[75] Dierker L., Swendsen J., Rose J., He J., Merikangas K., Tobacco Etiology Research Network (2012). Transitions to Regular Smoking and Nicotine Dependence in the Adolescent National Comorbidity Survey (NCS-A). Ann. Behav. Med..

[76] O’Loughlin J., Gervais A., Dugas E., Meshefedjian G. (2009). Milestones in the Process of Cessation Among Novice Adolescent Smokers. Am. J. Public Health.

[77] Zhan W., Dierker L.C., Rose J.S., Selya A., Mermelstein R.J. (2012). The Natural Course of Nicotine Dependence Symptoms Among Adolescent Smokers. Nicotine Tob. Res..

[78] Pampalon R., Hamel D., Gamache P., Philibert M.D., Raymond G., Simpson A. (2012). An Area-based Material and Social Deprivation Index for Public Health in Québec and Canada. Can. J. Public Health.

[79] Blakely T., Subramanian S.V., Oakes J.M., Kaufman J. (2006). Multilevel Studies. Methods in Social Epidemiology.

[80] Tabachnick B.G., Fidell L.S. (2013). Multilevel Linear Modeling. Using Multivariate Statistics.

[81] Chan J., Burnett T., Baillie R., Blomfield S., Cameron-Christie P., Dickson J., Fleishl W., Ghandi S., Gordon K., Heo J. (2014). Smoking in outdoor areas of bars and cafés: Large differences between midday and evening prevalences. Drugs Educ. Prev. Policy.

[82] International Agency for Research on Cancer (IARC) (2009). Handbooks of Cancer Prevention, Tobacco Control, Vol. 13: Evaluating the Effectiveness of Smoke-Free Policies.

[83] Cortese B.M., Uhde T.W., LaRowe S.D., Stein S.V., Freeman W.C., McClernon F.J., Brady K.T., Hartwell K.J. (2015). Olfactory Cue Reactivity in Nicotine-Dependent Adult Smokers. Psychol. Addict. Behav..

[84] Mead E.L., Rimal R.N., Ferrence R., Cohen J.E. (2014). Understanding the sources of normative influence on behavior: The example of tobacco. Soc. Sci. Med..

[85] Versace F., Engelmann J.M., Robinson J.D., Jackson E.F., Green C.E., Lam C.Y., Minnix J.A., Karam-Hage M.A., Brown V.L., Wetter D.W. (2014). Prequit FMRI responses to pleasant cues and cigarette-related cues predict smoking cessation outcome. Nicotine Tob. Res..

[86] Zhou X., Nonnemaker J., Sherrill B., Gilsenan A.W., Coste F., West R. (2009). Attempts to quit smoking and relapse: Factors associated with success or failure from the ATTEMPT cohort study. Addict. Behav..

[87] Trotter L., Wakefield M., Borland R. (2002). Socially cued smoking in bars, nightclubs, and gaming venues: A case for introducing smoke-free policies. Tob. Control.

[88] Gouvernement du Québec (2015). Bill 44: An Act to Bolster Tobacco Control.

[89] Novak S.P., Reardon S.F., Raudenbush S.W., Buka S.L. (2006). Retail Tobacco Outlet Density and Youth Cigarette Smoking: A Propensity-Modeling Approach. Am. J. Public Health.

[90] Burton S., Clark L., Jackson K. (2012). The association between seeing retail displays of tobacco and tobacco smoking and purchase: Findings from a diary-style survey. Addiction.

[91] Germain D., McCarthy M., Wakefield M. (2010). Smoker sensitivity to retail tobacco displays and quitting: A cohort study. Addiction.

[92] Henriksen L., Schleicher N.C., Feighery E.C., Fortmann S.P. (2010). A Longitudinal Study of Exposure to Retail Cigarette Advertising and Smoking Initiation. Pediatrics.

[93] Gauvin L., Robitaille E., Riva M., McLaren L., Dassa C., Potvin L. (2007). Conceptualizing and Operationalizing Neighborhoods: The Conundrum of Identifying Territorial Units. Can. J. Public Health.

[94] Cantrell J., Anesetti-Rothermel A., Pearson J.L., Xiao H., Vallone D., Kirchner T.R. (2015). The impact of the tobacco retail outlet environment on adult cessation and differences by neighborhood poverty. Addiction.

[95] Chaiton M., Mecredy G., Rehm J., Samokhvalov A.V. (2014). Tobacco retail availability and smoking behaviours among patients seeking treatment at a nicotine dependence treatment clinic. Tob. Induc. Dis..

[96] Riva M., Gauvin L., Barnett T.A. (2007). Toward the next generation of research into small area effects on health: A synthesis of multilevel investigations published since July 1998. J. Epidemiol. Community Health.

